# Supplementary material for: Investigating experiences of stigma and its impact on substance use recovery among residents of recovery residences in the United States: A scoping review protocol. BSGH 020
Source: PLoS One. 2024 Dec 2;19(12):e0309872. doi: 10.1371/journal.pone.0309872 (PMC11611164; doi:10.1371/journal.pone.0309872)
Supplement: Attachment — Submitted filename: renamed_8749c.docx [file pone.0309872.s001.docx]

# COMMENTS – Manuscript PONE-D-24-36007

Title: “Investigating experiences of stigma and its impact on substance use recovery among residents of recovery residences in the United States: A scoping review protocol. BSGH020”

**Important note: This review pertains only to ‘statistical aspects’ of the study and so ‘clinical aspects’ [like medical importance, relevance of the study, ‘clinical significance and** **implication(s)’ of the whole study, etc.] are to be evaluated [should be assessed] separately/independently. Further please note that any ‘statistical review’ is generally done under the assumption that study specific methodological [as well as execution] issues are perfectly taken care of by the investigator(s). This review is not an exception to that and so does not cover clinical aspects {however, seldom comments are made only if those issues are intimately / scientifically related & intermingle with ‘statistical aspects’ of the study}. Agreed that** **‘statistical methods’ are used as just tools here, however, they are vital part of methodology [and so should be given due importance]. I look at the manuscript in/with statistical view point, other reviewer(s) look(s) at it with different angle so that in totality the review is very comprehensive. However, there should be efforts from authors side to improve (may be by taking clues from reviewer’s comments). Therefore, please do not limit the revision only (with respect) to comments made here.**

**COMMENTS:** Though the study/manuscript is rather (almost) excellent, I have little/minor different opinion / observations/concerns or rather questions regarding (very) few issues (only two) which are given below:

Firstly, I noted that your ABSTRACT is very well drafted (in my opinion), but is ‘assay type’. It is preferable to divide the ABSTRACT with small sections like ‘Objective(s)’, ‘Methods’, ‘Results’, ‘Conclusions’, etc. which is an accepted practice of most of the good/standard journals [including this one, though ‘The PLoS One Guidelines to Authors’ did not specify an Abstract format, it is desirable]. It will definitely be more informative then, I guess, whatever the article type may be {though Section headings may differ for different Article Types [example: Study Protocol (for systematic review)]}.

Secondly (only a suggestion, may just consider, need not follow), I guess the title could be “The protocol of scoping* review on: Investigating experiences of stigma and its impact on substance use recovery among residents of recovery residences in the United States - BSGH020” instead of present “Investigating experiences of stigma and its impact on substance use recovery among residents of recovery residences in the United States: A scoping review protocol. BSGH020”.

*[A scoping review is a systematic method for identifying and mapping the available evidence on a topic, concept, or issue]

Will you please add ‘What is BSGH020 and why included it in the title. Purpose of publication of protocol of ‘A scoping review’ is also expected to be mentioned.

As pointed out in ‘important note’ above “This review pertains only to ‘statistical aspects’ of the study and so ‘clinical aspects’ should be assessed separately/independently. In my opinion, to make this article more acceptable (which is already of acceptable level), a small amount of re-vision may be needed.
