# Supplementary material for: Investigating experiences of stigma and its impact on substance use recovery among residents of recovery residences in the United States: A scoping review protocol. BSGH 020
Source: PLoS One. 2024 Dec 2;19(12):e0309872. doi: 10.1371/journal.pone.0309872 (PMC11611164; doi:10.1371/journal.pone.0309872)
Supplement: Attachment — Submitted filename: reviewers reponse letter (1).docx [file pone.0309872.s002.docx]

**Reponse to Reviewer**

Dear reviewer,

We have addressed all the recommendations provided by the reviewer. However, we would like to retain the title as it is: “Investigating experiences of stigma and its impact on substance use recovery among residents of recovery residences in the United States: A scoping review protocol. BSGH 020.” The title BSGH is the acronym for Behavioral, Sexual and Global Health Lab. Our manuscripts are abbreviated with the first letters of the lab title and numbers to keep track of our publications. We have independently addressed the statistical aspects separate from the clinical aspects in this protocol and highlighted these sections within the study document in yellow.

The following changes were recoomended and changed made:

1. The abstract be stratified by including sections (objectives, methods, results, conclusion).
2. Within the method section we have included a paragragh “(*We aim to employ statistical analysis as a component of our methodology, utilizing descriptive statistics to summarize stigma frequency and inferential methods to examine associations between stigma experiences and recovery outcomes. This approach is designed to ensure that the study produces reliable and meaningful data trends, contributing both to academic knowledge and practical implications for recovery residences*)”. This statement satifies reviewer comments **This review pertains only to ‘statistical aspects’ of the study and so ‘clinical aspects’ [like medical importance, relevance of the study, ‘clinical significance and** **implication(s)’ of the whole study, etc.] are to be evaluated [should be assessed] separately/independently***)”*
3. Within the strength and limitation section we have included a paragragh (*By taking an interdisciplinary approach, we hope this scoping review will offer statistical insights that contribute to a holistic understanding of stigma in recovery residences. We anticipate that integrating perspectives from clinical research would further enrich the applicability of these findings)*. This statement satifies reviewer comments **“(This review pertains only to ‘statistical aspects’ of the study and so ‘clinical aspects’ should be assessed separately/independently)”**

We hope these changes meet your recommendations and look forward to a positive response soon.

Sincerely

Osman Wumpini Shamrock PhD, MPhil

School of Nursing, University of Rochester

225 Crittenden Blvd, Rochester, NY, 14642

+1 (607) 761 8189
